# Supplementary material for: Phenolic Compounds Cannabidiol, Curcumin and Quercetin Cause Mitochondrial Dysfunction and Suppress Acute Lymphoblastic Leukemia Cells
Source: Int J Mol Sci. 2020 Dec 28;22(1):204. doi: 10.3390/ijms22010204 (PMC7795267; doi:10.3390/ijms22010204)
Supplement: Supplementary file 1 [file ijms-22-00204-s001.zip › ijms-1037361-Table S1.docx]

| **Table S1.** Characteristics of phenolic compounds exhibiting antileukemic activity | | | | | | |
| --- | --- | --- | --- | --- | --- | --- |
| **PHEN** | **Main Source*** | **Structure** | **Antileukemic effects** | **Working concentration** | **Experimental Model** | **References** |
| Aspirin | Synthetic | 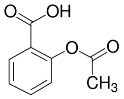 | ↓Viability ↑ proapoptotic Bcl-2 members, ↓ Mcl-1 ↓ risk of leukemia development | 0-10 mM | CLL  ALL (Jurkat)  AML / ALL (patients) | [1,2] |
| Cannabidiol | Cannabis Spp. | 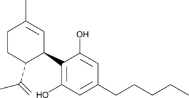 | ↓Viability, cell cycle arrest, cyt-c release, ↑ caspase, activation, ↑apoptosis, ↑ROS production, ↓ tumor burden | 0-100 μM | ALL (Jurkat, MOLT-4, CEM-CCFR)  ALL *xenograft* (EL-4) | [3-5] |
| Chlorogenic acid | Plum, coffee, cherry, peach, berries. | 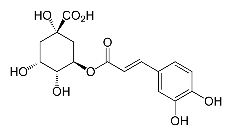 | ↓Proliferation, cell cycle arrest, ↑apoptosis, ↓ cell viability, ↑caspase activation, ↑ROS production, ↓ ∆ᴪm, cyt-c release, ↓tumor burden | 0-200 μM | APL (HL-60)  Promonocytic leukemia (U-937)  AML (K562)  ALL (MOLT-4, REH)  AML (patients)  CML *xenograft* (LAMA84) | [6-8] |
| Curcumin | Turmeric | 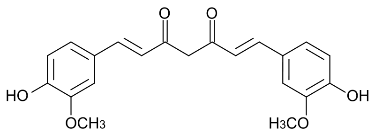 | ↓Cell viability, ↑ apoptosis, ↓ Bcl-2 members, cell cycle arrest, ↑caspase activation, ↓tumor burden , ↓ migration angiogenesis, ↑ ROS production | 0-100 μM | APL (HL-60)  AML (K562, MV4-11)  AML *xenograft*  ALL (REH, RS4;11)  CML (LAMA84) | [9-15] |
| Gallic acid | Nuts, blackberry, clove, wine | 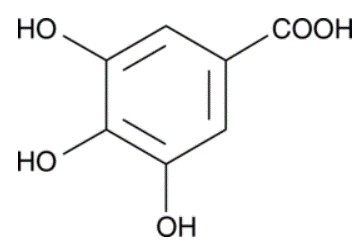 | ↓ ATP production ↓ oxygen consumption ↑ ROS, ↑ chemotherapy, ↓ viability, ↑ caspase activity, ↑ apoptosis, ↓ spleen / liver weight, ↓ Bcl-2, ↑ Bax, Cyt-c release, ↓∆ᴪm | 0-100 μM | AML (K562)  ALL (Jurkat)  *In vivo* (Murine leukemia, WEHI-231) | [16-20] |
| Methyl gallate | Terminalia, Bergenia, geranium. | 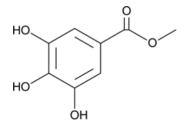 | ↓Proliferation, ↑ apoptosis, DNA fragmentation | 0-100 μM | ALL murine cell line (WEHI-231) | [20] |
| Protocatechuic acid | Wine, beer, date, sorghum, star anise, cardamom | 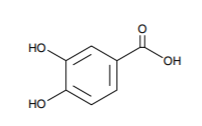 | ↓ Cell viability, DNA fragmentation | 0-2 mM | APL (HL60) | [21] |
| Quercetin | Wine, chocolate, berries, orange, oregano, apple, grapes, berries | 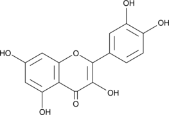 | ↑ Caspase activation, ↓ viability, ↓ mitochondrial activity, ↓ Bcl-2/Bcl-xL, ↓ Mcl-1, ↑ Bax, ↓ ∆ᴪm, ↑ apoptosis, cyt-c release, cell cycle arrest, ↓ tumor burden, ↓ proliferation, ↓ colony formation | 0-20 mM | ALL (MOLT-4, Jurkat)  CMML (P39)  AML (patients)  ALL (patients)  AML (K562)  APL (HL-60) | [22-26] |

Reference

1. Iglesias-Serret, D.; Piqué, M; Barragan, M.; Cosialls, A.M.; Santidrian, A.F.; Gonzales-Girones, D.M.; Coll-Mulet, L.; de Frias, M.; Pons, G.; Gil, J. Aspirin induces apoptosis in human leukemia cells independently of NF-κB and MAPKs through alteration of the Mcl-1/Noxa balance. *Apoptosis* **2010**, *15*, 219–229.
2. Weiss, J.R.; Baker, J.A; Baer, M.R.; Menezes, R.J.; Nowell, S.; Moysich, K.B. Opposing effects of aspirin and acetaminophen use on risk of adult acute leukemia. *Leuk. Res.* **2006**, *30*, 164–169.
3. Kalenderoglou, N.; Macpherson, T; Wright, K.R. Cannabidiol reduces leukemic cell size- but is it important? *Front. Pharmacol*. **2017**, *8*, 144.
4. McKallip, R.J.; Jia, W.; Schlomer, J.; Warren, J.W.; Nagarkatti, P.S.; Nagarkatti, M. Cannabidiol-induced apoptosis in human leukemia cells: A novel role of cannabidiol in the regulation of p22phox and Nox4 expression. *Mol. Pharmacol*. **2007**, *70*, 897–908.
5. Scott, K.A.; Dalgleish, A.G; Liu, W.M. Anticancer effects of phytocannabinoids used with chemotherapy in leukaemia cells can be improved by altering the sequence of their administration. *Int. J. Oncol*. **2017**, *51*, 369–377.
6. Liu, Y.L.; Zhou, C.Y.; Qiu, C.-H.; Lu, X.M.; Wang, Y.-T. Chlorogenic acid induced apoptosis and inhibition of proliferation in human acute promyelocytic leukemia HL-60 cells. *Mol. Med. Rep*. **2013**, *8*, 1106–1110.
7. Yang, J.-S.; Liu, C.-W.; Ma, Y.-S.; Weng, S.-W.; Tang, N.-Y.; Wu, S.-H.; Ji, B.-C.; Ma, C.-Y.; Ko, Y.-C.; Funayama, S.; et al. Chlorogenic acid induces apoptotic cell death in U937 leukemia cells through caspase- and mitochondria-dependent pathways. *In Vivo* **2012**, *26*, 971–978.
8. Bandyopadhyay, G.; Biswas, T.; Roy, K.C.; Mandal, S.; Mandal, C.; Pal, B. C.; Bhattacharya, S.; Rakshit, S.; Bhattacharya, D.K.; Chaudhuri, U.; et al. Chlorogenic acid inhibits Bcr-Abl tyrosine kinase and triggers p38 mitogen-activated protein kinase-dependent apoptosis in chronic myelogenous leukemic cells. *Blood* **2004**, *104*, 2514–2522.
9. Nagy, L.I.; Fehér, L.Z.; Szebeni, G.J.; Gyuris, M.; Sipos, P.; Alföldi, R.; Ozsvari, B.; Hackler, L.; Balázs, Árpád; Batar, P.; et al. Curcumin and its analogue induce apoptosis in leukemia cells and have additive effects with bortezomib in cellular and xenograft models. *Biomed. Res. Int.* **2015**, *2015*, 968981.
10. Martinez-Castillo, M.; Villegas-Sepulveda, N; Meraz-Rios, M. A.; Hernández-Zavala, A.; Berumen, J.; Coleman, M.A.; Orozco, L.; Cordova, E.J. Curcumin differentially affects cell cycle and cell death in acute and chronic myeloid leukemia cells. *Oncol. Lett*. **2018**, *15*, 6777–6783.
11. Yu, J.; Peng, Y.; Wu, L.-C.; Xie, Z.; Deng, Y.; Hughes, T.; He, S.; Mo, X.; Chiu, M.; Wang, Q.-E.; et al. Curcumin down-regulates DNA methyltransferase 1 and plays an anti-leukemic role in acute myeloid leukemia*. PLoS ONE* **2013**, *8*, e55934.
12. Mishra, D.; Singh, S.; Narayan, G. Curcumin induces apoptosis in pre-B acute lymphoblastic leukemia cell lines via PARP-1 cleavage. *Asian Pac. J. Cancer Prev*. **2016**, *17*, 3865–3869.
13. Taverna, S.; Giallombardo, M; Pucci, M.; Flugy, A.; Manno, M.; Raccosta, S.; Rolfo, C.; De Leo, G.; Alessandro, R. Curcumin inhibits *in vitro* and *in vivo* chronic myelogenous leukemia cells growth: A possible role for exosomal disposal of miR- 21. *Oncotarget* **2015**, *6*, 21918–21933.
14. Taverna, S.; Fontana, S.; Monteleone, F.; Pucci, M.; Saieva, L.; De Caro, V.; Giunta Cardinale, V.; Giallombardo, M.; Vicario, E.; Rolfo, C.; et al. Curcumin modulates chronic myelogenous leukemia exosomes composition and affects angiogenic phenotype via exosomal miR21. *Oncotarget* **2016**, *24*, 30420–30439.
15. Papiez, M.A.; Krzyściak, W; Szade, K.; Bukowska-Strakova, K.; Kozakowska, M.; Hajduk, K.; Bystrowska, B.; Dulak, J.; Jozkowiicz, A. Curcumin enhances the cytogenotoxic effect of etoposide in leukemia cell through induction of reactive oxygen species. *Drug Des. Devel. Ther*. **2016**, *10*, 557–570.
16. Gu, R.; Zhang, M; Meng, H.; Xu, D.; Xie, Y. Gallic acid targets acute myeloid leukemia via Akt/mTOR-dependent mitochondrial respiration inhibition. *Biomed. Pharmacother*. **2018**, *105*, 491–497.
17. Sourani, Z.; Pourgheysari, B.; Beshkar, P.; Shirzad, H.; Shirzad, M. Gallic acid inhibits proliferation and induces apoptosis in lymphoblastic leukemia cell line (C121). *Iran J. Med. Sci*. **2016**, *41*, 525–530.
18. Ho, C.-C.; Lin, S.-Y.; Yang, J.-S.; Liu, K.-C.; Tang, Y.-J.; Yang, M.-D.; Chiang, J.-H.; Lu, C.-C.; Wu, C.-L.; Chiu, T.-H.; et al. Gallic acid inhibits murine leukemia WEHI-3 cells in vivo and promotes macrophage phagocytosis. *In Vivo* **2009**, *23*, 409–414.
19. Reddy, T.C.; Reddy, D.B; Aparna, A; Arunasree, K.M.; Gupta, G.; Achari, C.; Reddy, G.V.; Lakshmipathi, V.; Subramanyam, A.; Reddanna, P. Anti-leukemic effects of gallic acid on human leukemia K562 cells: Downregulation of COX-2, inhibition of BCR/ABL kinase and NF-kB Inactivation. *Toxicol. In Vitro* **2012**, *26*, 396–405.
20. Serrano, A.; Palacios, C; Roy, G.; Cespón, C.; Villar, M. L.; Nocito, M.; González-Porque, P. Derivatives of gallic acid induce apoptosis in tumoral cell lines and inhibits lymphocyte proliferation*. Arch. Biochem. Biophys.* **1998**, *350*, 49–54.
21. Tseng, T.-H.; Kao, T.-W.; Chu, C.-Y.; Chou, F.-P.; Lin, W.-L.; Wang, C.-J. Induction of apoptosis by hibiscus protocatechuic acid in human leukemia cells via reduction of retinoblastoma (RB) phosphorylation and Bcl-2 expression. *Biochem. Pharmacol*. **2000**, *60*, 307–315.
22. Maso, V.; Calgarotto, A.K.; Franchi, G.C.; Nowill, A.E.; Filho, P.L.; Vassallo, J.; Saad, S.T.O. Multitarget effects of quercetin in leukemia. *Cancer Prev. Res*. **2014**, *7*, 1240–1250.
23. Larocca, L.M.; Teofili, L; Sica, S.; Plantelli, M.; Maggiano, N.; Leone, G.; Ranelletti, F. O. Quercetin inhibits the growth of leukemic progenitors and induces the expression of transforming growth factor-beta 1 in these cells. *Blood* **1995**, 3654–3661.
24. Kawahara, T.; Kawaguchi-Ihara, N.; Okuhashi, Y.; Itoh, M.; Nara, N.; Tohda, S. Cyclopamine and quercetin suppress the growth of leukemia and lymphoma cells. *Anticancer Res*. **2009**, *29*, 4629–4632.
25. Calgarotto, A.K.; Maso, V.; Junior, G.C.F.; Nowill, A.E.; Filho, P.L.; Vassallo, J.; Saad, S.T.O. Antitumor activities of quercetin and green tea in xenografts of human leukemia HL60 cells. *Sci. Rep*. **2018**, *8*, 1–7.
26. Rothwell, J.A.; Perez-Jimenez, J.; Neveu, V.; Medina-Remón, A.; M’Hiri, N.; García-Lobato, P.; Manach, C.; Knox, C.; Eisner, R.; Wishart, D.S.; et al. Phenol-Explorer 3.0: A major update of the phenol-explorer database to incorporate data on the effects of food processing on polyphenol content. *Database* **2013**, *2013*, bat070.
