# Supplementary material for: Phenolic Compounds Cannabidiol, Curcumin and Quercetin Cause Mitochondrial Dysfunction and Suppress Acute Lymphoblastic Leukemia Cells
Source: Int J Mol Sci. 2020 Dec 28;22(1):204. doi: 10.3390/ijms22010204 (PMC7795267; doi:10.3390/ijms22010204)
Supplement: Supplementary file 1 [file ijms-22-00204-s001.zip › ijms-1037361-Table S2.docx]

**Table S2.** List of chemicals, cell lines, software, and digitalized molecular structures.

| **Material** | **Source** | **ID** |
| --- | --- | --- |
| **Reagents** | | |
| Cannabidiol (CBD) | Cayman Chemical | 90081, CAS N°13956-29-1 |
| Aspirin | Sigma-Aldrich | A2093 |
| Curcumin | Sigma-Aldrich | C7727 |
| Tetramethylrhodamine, Ethyl Ester, Perchlorate (TMRE) | Thermo Fisher Scientific | #T669 |
| Dichlorodihydrofluorescein diacetate (DCFHDA) | Sigma-Aldrich | #D6883 |
| Rhod-2, AM, cell permeant | Thermo Fisher Scientific | #R1244 |
| Phorbol 12 myristate 13 acetate (PMA) | Sigma-Aldrich | #P8139 |
| Chlorogenic acid | Cayman Chemical | #70930 |
| Gallic acid | Cayman Chemical | #11846 |
| Methyl gallate | Cayman Chemical | #19951 |
| Protocatechuic acid | Cayman Chemical | #14916 |
| Quercetin | Cayman Chemical | #10005169 |
| **Comercial assays** | | |
| *In vitro* toxicology assay kit, resazurin based | Sigma-Aldrich | 263-718-5 |
| **Data from digital sources** | | |
| hVDAC1 (human voltage-dependent anion channel) structure | PDB | 2JK4 |
| Aspirin | Pubchem (NIH) | 2244 |
| Cannabidiol (CBD) | Pubchem (NIH) | 644019 |
| Chlorogenic acid | Pubchem (NIH) | 1794427 |
| Curcumin | Pubchem (NIH) | 969516 |
| Gallic acid | Pubchem (NIH) | 370 |
| Methyl gallate | Pubchem (NIH) | 7428 |
| Protocatechuic acid | Pubchem (NIH) | 72 |
| Quercetin | Pubchem (NIH) | 5280343 |
| **Experimental models (cell lines)** | | |
| Human: Jurkat, clone E6-1 (male, 14 yrs) | ATCC | TIB-152 |
| **Software and Algorithms** | | |
| ZEN lite | Zeiss | https://www.zeiss.com/microscopy/int/products/microscope-software/zen-lite.html |
| ImageJ | NIH | https://imagej.nih.gov/ij/ |
| Molegro virtual docker | CLC bio company | https://www.qiagenbioinformatics.com/products/molegro-virtual-docker/latest-improvements/ |
| Graphpad prism | Graphpad Software Inc. | www.graphpad.com |
| FlowJo | Becton Dickinson | https://www.flowjo.com/solutions/flowjo/downloads |
| FL-solutions | Hitachi | https://www.hitachi-hightech.com/us/product_detail/?pn=ana-f7000 |
