# Supplementary material for: Phenolic Compounds Cannabidiol, Curcumin and Quercetin Cause Mitochondrial Dysfunction and Suppress Acute Lymphoblastic Leukemia Cells
Source: Int J Mol Sci. 2020 Dec 28;22(1):204. doi: 10.3390/ijms22010204 (PMC7795267; doi:10.3390/ijms22010204)
Supplement: Supplementary file 1 [file ijms-22-00204-s001.zip › Figure s1.docx]

**
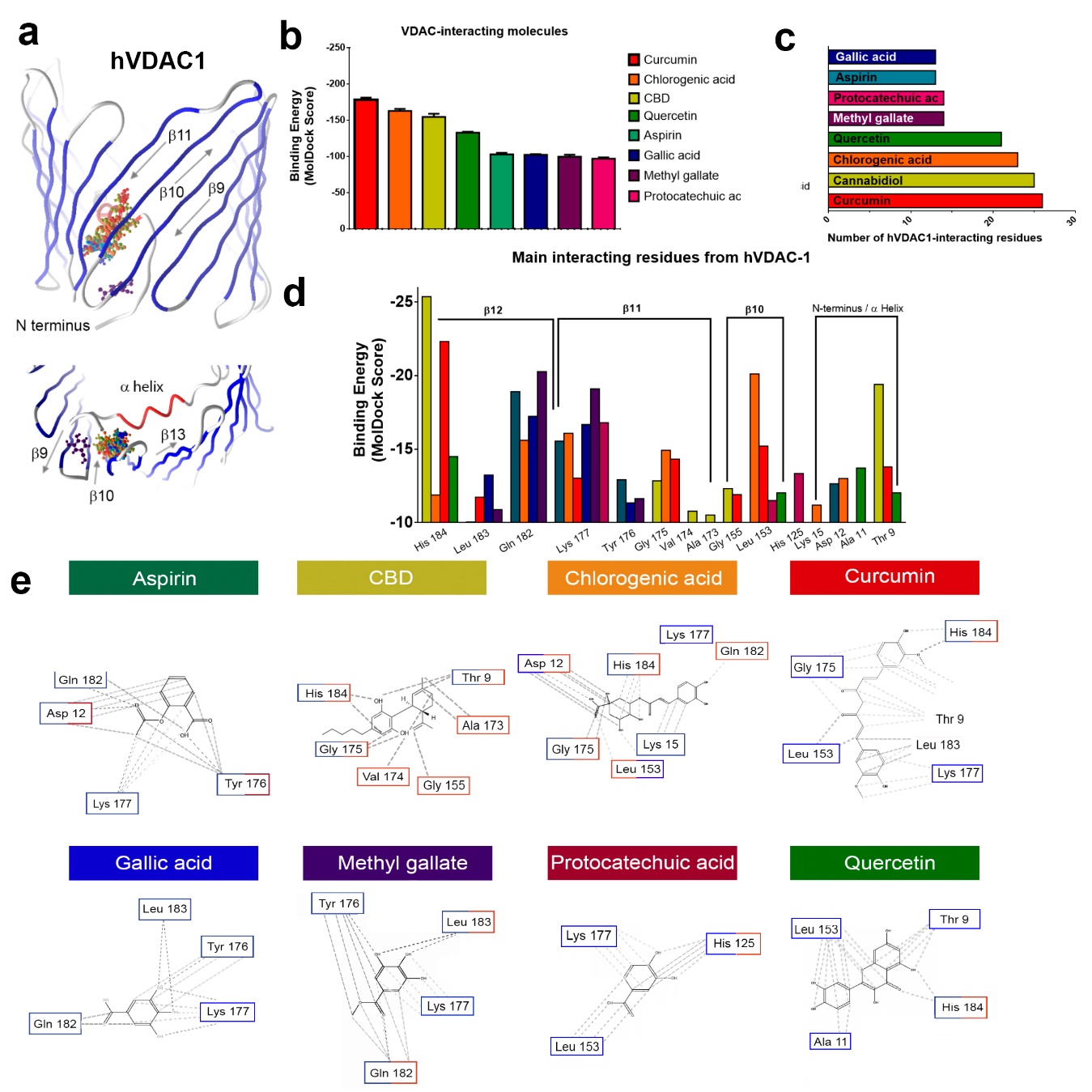
**

**Supplementary figure 1**. Docking analysis of possible molecular interactions of phenols with hVDAC1. (a) Most phenols are predicted to interact with hVDAC1 within a restricted area. hVDAC1 (2JK4) structure from Protein Data Bank (PDB) and the most favorable prediction for each phenols (PubChem identifier: Aspirin, 2244; CBD, 644019; Curcumin, 969516; Chlorogenic acid, 1794427; Gallic acid, 370; Methyl gallate, 7428; Protocatechuic acid, 72; Quercetin, 5280343), is depicted (color coded). All phenols are predicted to interact within a conserved pocket or cavity, which includes the N-terminal α helix and β9-13 strands (b) Total binding energy for 5 best binding positions for each phenol, mean ± SD. (c) The average number of interacting hVDAC1 residues with each phenol. (d) Contribution of individual amino acid residues to phenols binding. The energy of -10 (MolDock Score) was taken as a threshold. (e) Co-ordination of phenols by respective hVDAC1 residues, blue squares represent steric interaction, whereas red boxes indicate the hydrogen bond. Note that efficient phenols, CBD, curcumin and quercetin displayed a high overall binding affinity and had the strongest preference for the two specific residues, His 184 (pore wall) and Thr 9 (α helix in the N-terminus).
