# Supplementary figures and images for: Phenolic Compounds Cannabidiol, Curcumin and Quercetin Cause Mitochondrial Dysfunction and Suppress Acute Lymphoblastic Leukemia Cells
Source: Int J Mol Sci. 2020 Dec 28;22(1):204. doi: 10.3390/ijms22010204 (PMC7795267; doi:10.3390/ijms22010204)

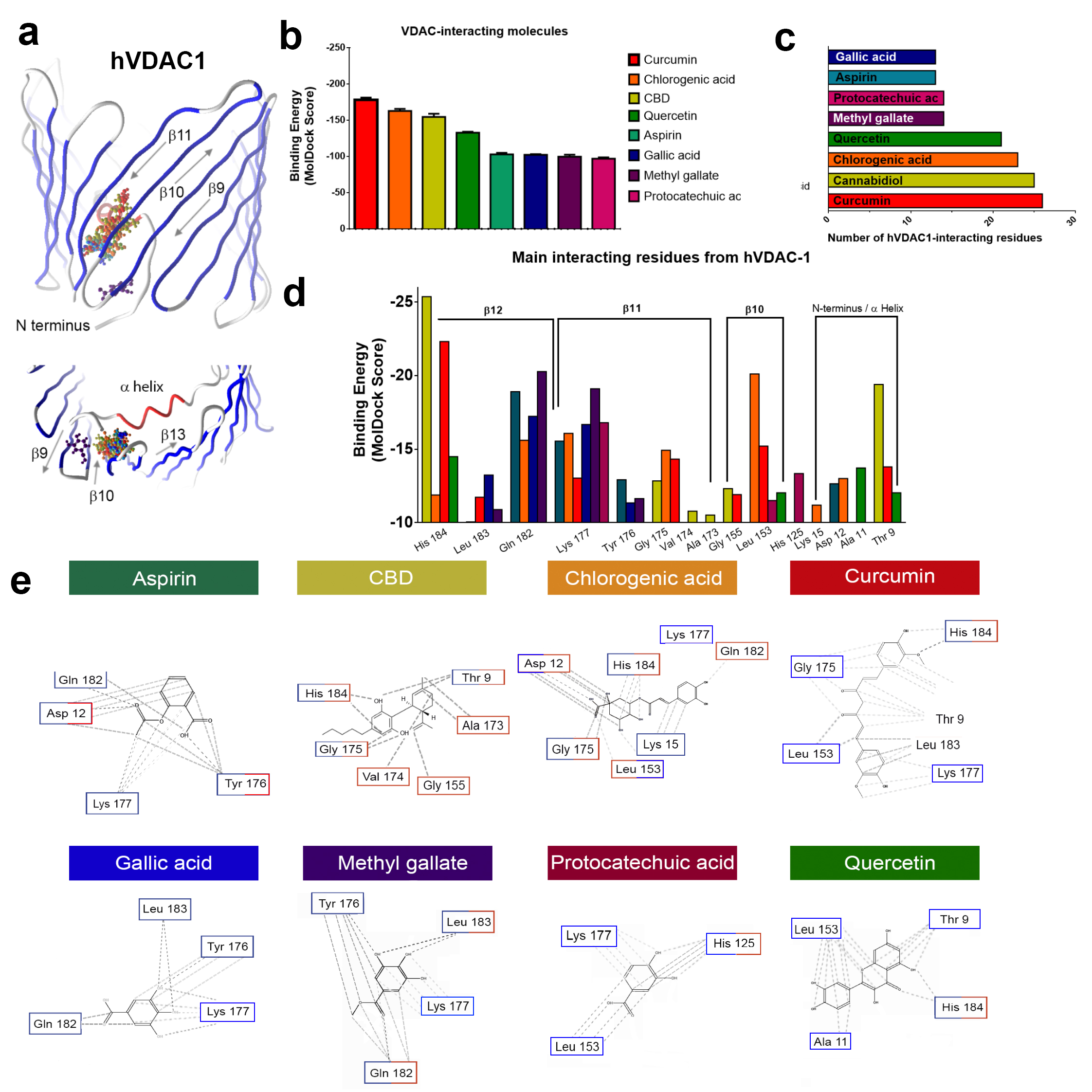

Supplement: Supplementary file 1 [file ijms-22-00204-s001.zip › Figura s1.jpg]
